# Adrenal Extract in Graves’ Disease

**Published:** 1913-11

**Authors:** 


ADRENAL EXTRACT IN GRAVES' DISEASE. C. E. H. Warren, *Lancet,* March 18, 1913, reports a case greatly improved. We have personally used this method in about ten cases, dating back about ten years, with improvement in all except one case in which high-frequency cataphoresis was attempted instead of internal administration. This case, however, was very unruly and the duration of treatment was insufficient. One of the cases resulted in the complete disappearance of an apple-sized goitre, which caused the patient much mortification. She has for some years been able to dispense with laces, etc., at the neck and can wear costumes that would be indecent in a better developed person.

